# Competitive Exclusion and Coexistence of Pathogens in a Homosexually-Transmitted Disease Model

**DOI:** 10.1371/journal.pone.0016467

**Published:** 2011-02-15

**Authors:** Caichun Chai, Jifa Jiang

**Affiliations:** 1 Caichun Chai College of Statistics and Applied Mathematics and Institute of Applied Mathematics, Anhui University of Finance and Economics, Bengbu, Anhui, China; 2 Jifa Jiang Mathematics and Science College, Shanghai Normal University, Shanghai, Shanghai, China; Dana–Farber Cancer Institute, United States of America

## Abstract

A sexually-transmitted disease model for two strains of pathogen in a one-sex, heterogeneously-mixing population has been studied completely by Jiang and Chai in (J Math Biol 56:373–390, 2008). In this paper, we give a analysis for a SIS STD with two competing strains, where populations are divided into three differential groups based on their susceptibility to two distinct pathogenic strains. We investigate the existence and stability of the boundary equilibria that characterizes competitive exclusion of the two competing strains; we also investigate the existence and stability of the positive coexistence equilibrium, which characterizes the possibility of coexistence of the two strains. We obtain sufficient and necessary conditions for the existence and global stability about these equilibria under some assumptions. We verify that there is a strong connection between the stability of the boundary equilibria and the existence of the coexistence equilibrium, that is, there exists a unique coexistence equilibrium if and only if the boundary equilibria both exist and have the same stability, the coexistence equilibrium is globally stable or unstable if and only if the two boundary equilibria are both unstable or both stable.

## Introduction

An important principle in theoretical biology is that of competitive exclusion: no two species can forever occupy the same ecological niche. Classifications on the meaning of competitive exclusion and niche have been central to theoretical ecology [1]–[4]. On the other hand, biologists and mathematical modelers have long been concerned with the evolutionary interactions that result from changing host and pathogen populations. Continuous advances in biology and behavior have brought to the forefront of research the importance of their role in disease dynamics [5]–[17]. Sexually transmitted diseases, such as gonorrhea have incredibly high incidences throughout the world, providing the necessary environment and opportunities for the evolution of new strains(see [18] and the references therein). The coexistence of gonorrhea strains has become an increasingly serious problem. Understanding the mechanisms that lead to coexistence or competitive exclusion is critical to the development of disease management strategies, as well as to our understanding of STD dynamics.

In previous papers [18], [19], they have shown that coexistence of multiple strains is not possible in a heterosexually-active homogenous population where individuals have the same mean behavior by investigating SIS STD models and establishing that such populations are unable to support multiple strains. However, using simple heterosexual mixing models, Castillo-Chaves et al. [20], [21] have shown that heterogeneity(behavioral or genetically or a combination of both) of one sex population(the female population) is enough to maintain heterogeneity and to lead possible coexistence of multiple strains. Chai [22] and Qiu [23] has given the completely classification for this model. Li et al. [24] have determined what is the minimum level of heterogeneity required to support multiple strains to coexist. They formulated and analyzed a one-sex, SIS STD model with two competing strains under the same assumptions. Furthermore, in [25], we have presented a thorough classification of dynamics for this model in terms of the first and the second so called reproductive numbers, and discussed the biological meaning of our results in the finally.

This paper focus on the dynamics of sexually transmitted pathogens in a homosexually active population, where populations are divided into three groups based on their susceptibility to infection(colonization) by two distinct pathogenic strains of an STD. It is assumed that a host cannot be invaded simultaneously by both disease agents(that is, there is no superinfection) and that when symptoms appear-a function of pathogen, strain, virulence, and an individual's degree of susceptibility-then individuals are treated and/or recover.

## Methods

Let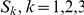, denote the susceptibles with sexual activity 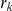, which is the number of contacts per individual in group 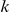 per unit of time, and use 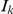 and 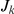 to denote the infectives with sexual activity 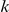 and infected by strain 1 and strain 2, respectively. The dynamics of the disease transmission then is described by the following equations:
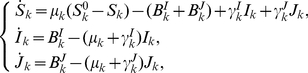
(1)where
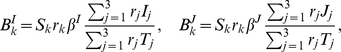
are the rates of incidence with 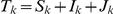 being the population size of group 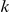, 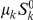 are the constant input flows entering the sexually active sub-populations, 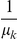 are the average sexual life spans for people in group 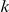, 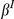 and 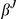 are the transmission probabilities per contact with individuals infected by strains 1 and 2, respectively, and 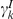 and 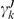 are the rates of recovery for classes 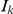 and 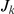, respectively. It is assumed that people with different sexual activity having different rates of recovery as highly sexually-active individuals may have health examinations more frequently.

The limiting system of (1) is
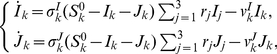
(2)where
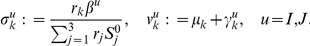



Set
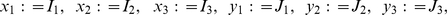


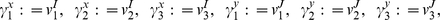


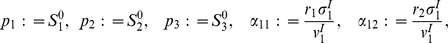


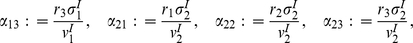


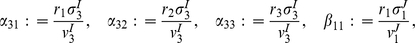


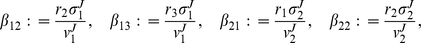


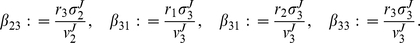
Then
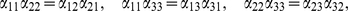


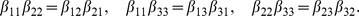
With these notations, the system (2) can be rewritten into the following compact form:
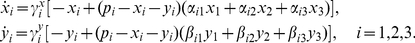
(3)


Note that 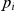 is the total population of group 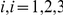. Throughout this paper will consider only the dynamics of (3) in 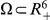, where
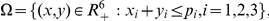
and 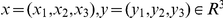. Let 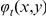 denote the solution flow generated by (3). It is not difficult to see that the flow is positively invariant in 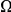.

For two vectors 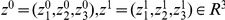, define the vector order as follows:
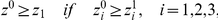
and also define the type-K order in 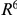 in the sense that
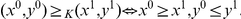
The Jacobian-matrix at each point 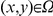 has the form
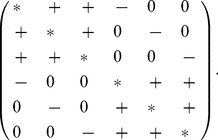
(4)It follows from Smith [26] that the flow 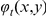 is type-K monotone in the sense that
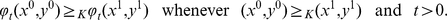



## Discussion

Next, we consider the necessary thresholds and the stability of the infection-free state, established the principle of competitive exclusion and coexistence for SIS models with heterogeneous mixing.

### Thresholds

The linearization about the infection-free equilibrium of (3) is
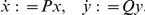
where
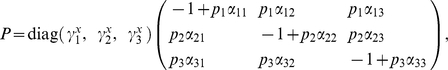


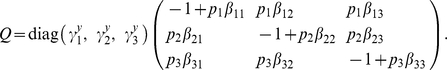
Now we define the reproductive numbers
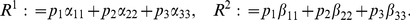
(5)Hence, by calculation, it follows from M-matrix theory [27], if 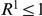 and 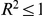, then the origin is locally asymptotically stable. If 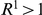 or 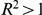, the infection-free equilibrium is unstable.

As in [24], it can be shown that the locally stable infection-free equilibrium and the locally stable boundary equilibrium associated with model (3), which will be studied in the following section, are globally stable. We only state the results as follows and omit the details. The interested reader is referred to [24].

#### Lemma 1

*Let* 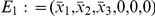 *and* 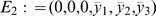 *be equilibria of (3), where* 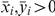 *, if* 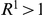 *and* 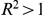 *;* 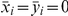 *, if* 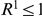 *and* 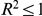 *,* 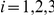 *. Let* 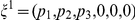 *and* 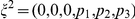 *. Then*

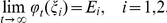



In summary, we state the threshold conditions for the disease as follows.

#### Theorem 1

*Let the reproductive number* 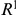 *and* 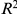 *be defined in (5). Then, if* 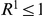 *and* 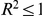 *, the infection-free equilibrium is globally asymptotically stable so that the epidemic goes extinct regardless of the initial levels of infection. If* 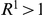 *or* 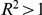 *, then the infection-free equilibrium is unstable and the epidemic spreads in the population*.

### The computation of boundary equilibria

Let 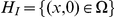 and 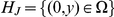. Then 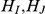 are invariant for (3). The subsystems on 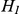 and 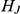 are
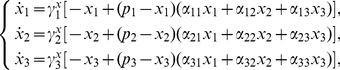
(3)_{\it I})and
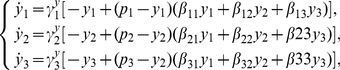
(3)_{\it J})respectively.

Following Smith [28], both 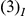 and 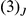 are strongly concave. From [28] it follows that the origin is globally asymptotically stable, or there is exists and equilibrium 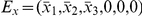 with 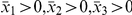 such that it is globally asymptotically stable in 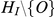. Moreover, 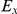 is also linearly stable, that is,
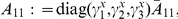


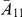
 has the following form
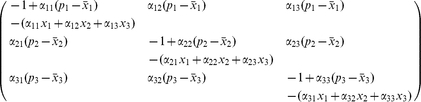
is stable matrix.

From Theorem 1, if 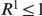, then the origin is globally asymptotically stable in 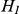, otherwise, 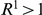, 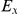 exists. Next, we discuss the computation for 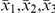 for the case 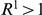. Make the transformation
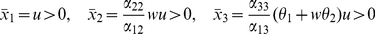
(6)where
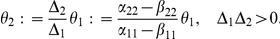
Then 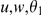 satisfy the equations
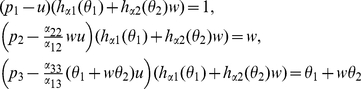
(7)where
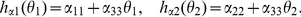
(8)By (7), we have
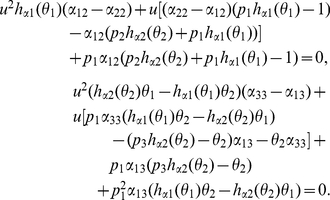
(9)


Now, we assume that 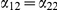 and 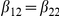, (9) is equivalent to
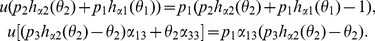
(10)


Let
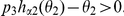



Solving 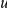 in (10),we get that
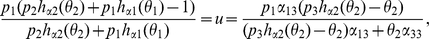
which implies that 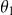 must be the positive root of
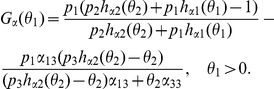
Let
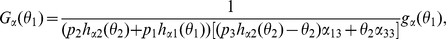
where
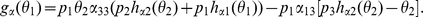
Since 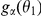 is a quadratic function in 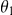 with 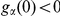 and the coefficient of second order positive, there exists a unique real number 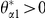 such that
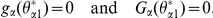



In addition
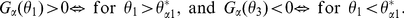
(11)


Similarly, the origin is globally asymptotically stable in 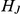 if 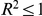. Otherwise, if 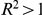, then 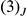 has an equilibrium 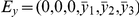 with 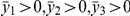 such that it is globally asymptotically stable in 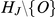. Moreover, 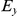 is also linearly stable, that is,
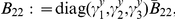


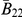
 has the following from
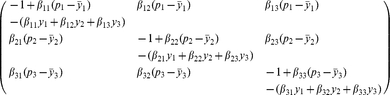
is stable. The positive components 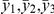 can be calculated by
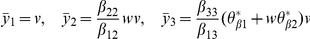
where
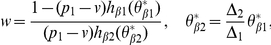
and 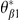 is the unique positive root for
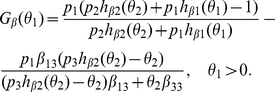
(12)We have the following inequalities
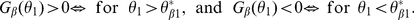
(13)All above computation results will be very useful in the classification for various dynamical behavior. Before finishing this section, we present a result for (3) that is easily obtained by the theory of monotone dynamical systems.

#### Theorem 2

(i) *The infection-free equilibrium* 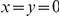 *is globally asymptotically stable if and only if the reproductive numbers* 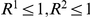 *.*

(ii) *If* 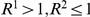, *then* 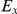 *is globally asymptotically stable in* 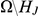.

(iii) *If* 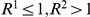, *then* 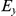 *is globally asymptotically stable in* 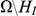.

### The stability of boundary equilibria

First, in the case that either 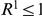 or 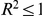, Theorem 2 tell us that the global behavior for (3) is clear. So it suffices to consider the case both 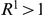 and 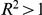.

Let
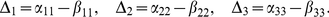
(14)


From now on, we discuss the stability of the boundary equilibrium 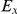.

The Jacobian matrix 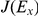 of (3) at 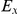 takes the form
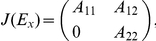
where 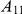 is a stable matrix in the above section and
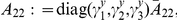


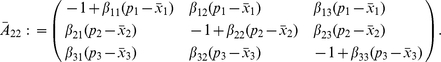
It follows from [27] or Theorem 2.3 in [26] that the stability for the matrices 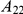 and 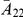 is all the same. By calculation,
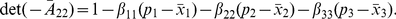
(15)From the first equation of 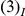 and (6) we get that
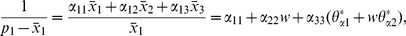
(16)and by (7), we have
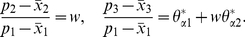
(17)It deduces from (16) and (17) that
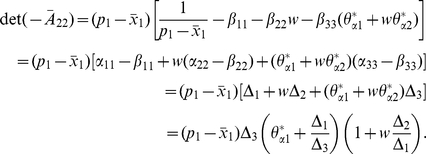



Then, from M-matrix theory [27], it is easy to get that 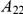 is stable (unstable) if and only if 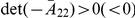, that is, 
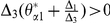
, where 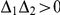 in the above section.

Then we have the results as follows:

#### Theorem 3

*Let* 
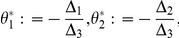
 *and* 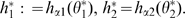.

*(I)* 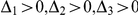, 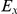 *is stable*;

*(II)* 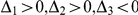, 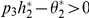 and


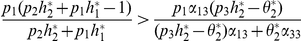
, 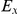 *is stable*;

*(III)* 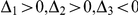, 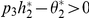 *and*


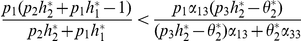
, 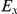 *is unstable.*

In a quite similar way, we can discuss the stability for the boundary equilibrium 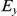, its stability is completely determined by the determinant of the matrix
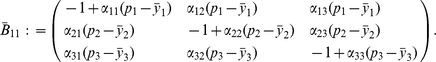
The computation shows that
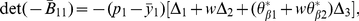
(18)where 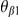 is given in (12) and (13).

Observing that 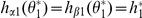 and 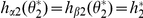, we get the following stability results from (18):

#### Theorem 4

*The stability for* 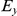 *is confirmed by using (18) as follows:*

*(I)* 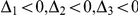, 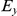 is stable;

(II) 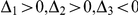, 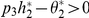 and


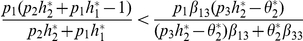
, 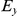 is stable;

*(III)* 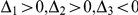, 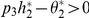 and


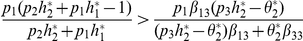
, 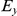 is unstable.

#### Remark 1

*In Theorem 3 and Theorem 4, we only give the results in this case* 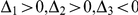 *. The other cases can be considered analogously by changing the relevant parameters.*

Let 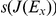 and 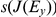 denote the largest real part of its eigenvalues respectively, which is an eigenvalue for 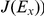 and 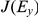 respectively by Perron-Frobenius theory [27].

#### Remark 2

*Suppose that* 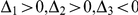 *. Then* 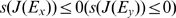 *implies that* 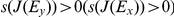.

Proof Suppose that 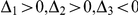. We have
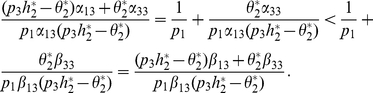
Then
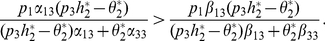
(19)The discussion in the above has shown that 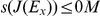 is equivalent to
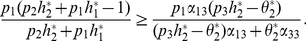
(20)(19) and (20) deduce that
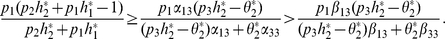
By Theorem 4, 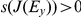.

The other cases can be considered analogously.

#### Remark 3

*Suppose* 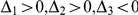 *and* 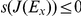 *. If there is no positive equilibrium in* 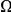 *, then* 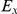 *is globally asymptotically stable in* 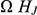 *. Similar result holds for* 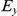.

### The existence of endemic equilibrium

It follow from Theorem 2 that one of the necessary conditions for existence of positive equilibrium is that 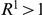 and 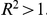


Now, let we assume 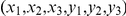 is a positive equilibrium for (3), and set
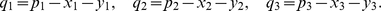



Then 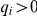 for 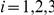 and 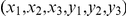 satisfies
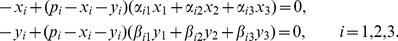
(21)


Thus
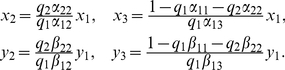
(22)


Substituting (22) into (21) yields
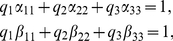
(23)hence
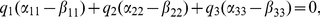
(24)which implies by 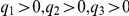 that either
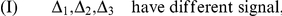
or
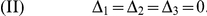



In order to study the existence of positive equilibrium, we only need to consider the case (I) and (II). Suppose first the former holds. Without loss of generality, we assume that 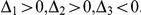 Let
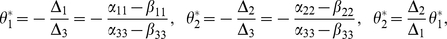
then
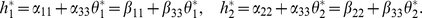



By (24), we have
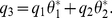
(25)


Substituting (25) and (23) into (22), we conclude that such a positive equilibrium must have the form
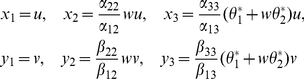
(26)where
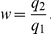



Substituting (26) into (21), we obtain the equations for 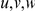 in the form
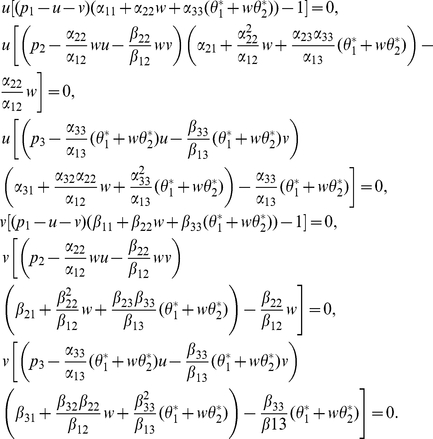



By calculation, we have
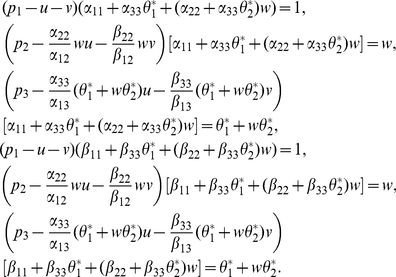
(27)


Notice that
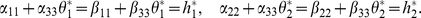



Then, (27) is reduced to the system
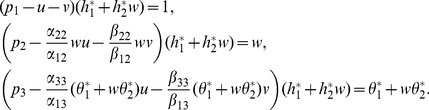
(28)


By (28), we have
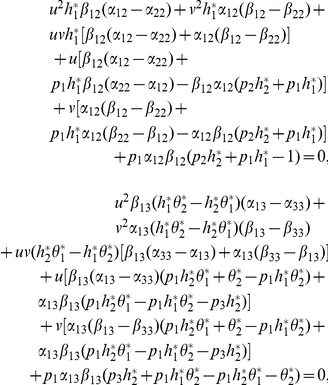
that is
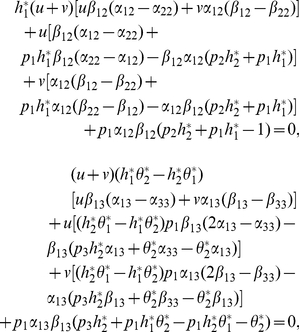
(29)


Notice that
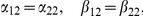
then
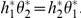



From (29), we obtain
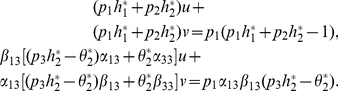
(30)


Then, (30) has a unique positive solution if and only if
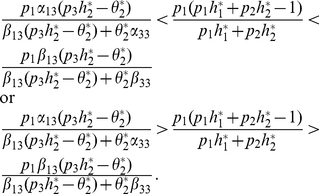
(31)


Moreover, we have the result as follows:

#### Theorem 5

*If* 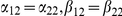 *, and* 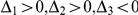 *. System (3) has a unique positive solution if and only if the following conditions is satisfied:*

*(H1)* 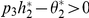 *and*

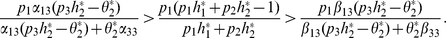



*(H2)* 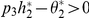 *and*

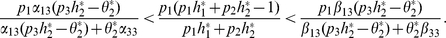



It follows from (4) and Smith [26] that (3) is type-K monotone system, hence 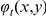 tends to an equilibrium as 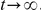 Then we can give stability conditions for the positive coexistence equilibrium as follows.

#### Theorem 6

*The positive coexistence equilibrium is stable if*

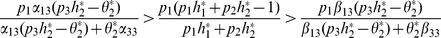

*and is unstable if*

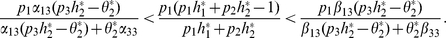



It remains to consider the case 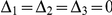. In this case, it is easy to verify 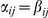 for 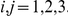 Thus 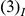 and 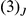 are the same. Let 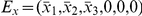. Then 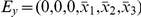. Set
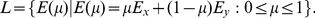
(32)


Then a straight proof by using 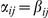 shows that all points in segment L are nontrivial equilibria for (3).

#### Theorem 7

*Suppose that*

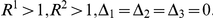



*Then nontrivial equilibria set for (3) is L. Moreover, for any* 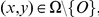 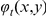 *tends to an equilibrium in L as* 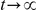.

The proof refer to the proof of Theorem 4.2 in [25].

## Results

In this article, we have given the stability analysis of the nontrivial boundary equilibria and the positive coexistence equilibrium. Our results can be summarized as the following:

*System (3) (and hence (1))has a unique positive coexistence equilibrium if and only if the two nontrivial boundary equilibria have the same stability. (Both are stable or unstable.) The positive coexistence equilibrium is stable if the boundary equilibria are both unstable. In this case the positive coexistence is a globally attractor. The positive coexistence equilibrium is unstable if and only if the boundary equilibria are both stable. The sufficient and necessary conditions for both boundary equilibria to be stable (unstable) and hence for the positive coexistence equilibrium to be unstable (stable) are given by (H1), (H2) in Theorem 5. Furthermore, if there is no coexistence equilibrium, then the locally stable boundary equilibrium, if it exist, is also globally stable.*

In the paper [25], we have given the biological meanings for our results. The biological meanings for the results in this paper which can be given in the same way. The interested reader is referred to [25].
